# Highly Enhanced H_2_ Sensing Performance of Few-Layer MoS_2_/SiO_2_/Si Heterojunctions by Surface Decoration of Pd Nanoparticles

**DOI:** 10.1186/s11671-017-2335-y

**Published:** 2017-10-17

**Authors:** Lanzhong Hao, Yunjie Liu, Yongjun Du, Zhaoyang Chen, Zhide Han, Zhijie Xu, Jun Zhu

**Affiliations:** 1 College of Science, China University of Petroleum, Qingdao, Shandong 266580 China; 2 State Key Laboratory of Electronic Thin Films and Integrated Devices, University of Electronic Science and Technology of China, Chengdu, 610054 China

**Keywords:** Molybdenum disulfide, Heterojunction, Sensing, Sputter, Surface

## Abstract

A novel few-layer MoS_2_/SiO_2_/Si heterojunction is fabricated via DC magnetron sputtering technique, and Pd nanoparticles are further synthesized on the device surface. The results demonstrate that the fabricated sensor exhibits highly enhanced responses to H_2_ at room temperature due to the decoration of Pd nanoparticles. For example, the Pd-decorated MoS_2_/SiO_2_/Si heterojunction shows an excellent response of 9.2 × 10^3^% to H_2_, which is much higher than the values for the Pd/SiO_2_/Si and MoS_2_/SiO_2_/Si heterojunctions. In addition, the H_2_ sensing properties of the fabricated heterojunction are dependent largely on the thickness of the Pd-nanoparticle layer and there is an optimized Pd thickness for the device to achieve the best sensing characteristics. Based on the microstructure characterization and electrical measurements, the sensing mechanisms of the Pd-decorated MoS_2_/SiO_2_/Si heterojunction are proposed. These results indicate that the Pd decoration of few-layer MoS_2_/SiO_2_/Si heterojunctions presents an effective strategy for the scalable fabrication of high-performance H_2_ sensors.

**Electronic supplementary material:**

The online version of this article (10.1186/s11671-017-2335-y) contains supplementary material, which is available to authorized users.

## Background

As a clean and abundant energy source, hydrogen (H_2_) has been utilized in various kinds of fuel cells. At the same time, H_2_ is a tasteless, colorless, and explosive gas, which can cause some safety concerns [1]. For the safe operation, H_2_ sensors are thus crucial to detect and monitor H_2_ leaks in real time. Presently, metal oxide sensors are effective for the detection of H_2_ [2–5]. However, metal oxide-based H_2_ sensors require high operation temperature (~ 150 °C), which can pose a risk for safety itself since H_2_ is highly flammable. In this regard, it is highly desirable to synthesize novel sensitive materials to develop reliable H_2_ sensors which can operate at room temperature (RT).

Molybdenum disulfide (MoS_2_), as one typical candidate of graphene analogues and a member of the transition metal dichalcogenides (TMDs), has recently drawn tremendous attention due to its excellent properties [6–10]. Structurally, each MoS_2_ unit layer is consisted of covalently bonded Mo–S atoms and the neighbor layers attach each other by van der Waals forces. These characteristics, on the one hand, promise two-dimensional (2D) MoS_2_ a high surface-to-volume ratio. On the other hand, MoS_2_ can be exfoliated into monolayer or few layers easily due to the weak van der Waals forces between atomic layers. Even with the desirable qualities of MoS_2_ for a variety of applications, the fabrication of large area, high-quality MoS_2_ ultra-thin films remains a challenge to this date. Conventional approaches, such as mechanical exfoliation [11–13], yield localized layered flakes that are not scalable for large area device applications. In recent years, chemical vapor deposition has been explored for producing large-area MoS_2_ mono/few-layer films [14–16]. However, this technique requires high process temperatures in the range of 800–1000 °C which could cause serious volatility of the sulfur in the layers and the diffusion at the interface. Thus, it is necessary to develop alternative synthesis methods capable of growing large-area MoS_2_ ultra-thin films. Recently, physical vapor deposition, mainly including magnetron sputtering technique and pulsed layer deposition [17–22], is proved to be another effective approach to realize the growth of wafer-scale MoS_2_ mono/few-layer films at much lower growth temperature of around 300 °C. The results demonstrate that the sputtered few-layer MoS_2_ films exhibit remarkable transporting characteristics, such as a high mobility of ~ 181 cm^2^/Vs and a large current on/off ratio of ~ 10^4^ [20].

Based on the large surface-to-volume ratio and excellent semiconducting transporting properties, mono/few-layer MoS_2_ films are expected to be potential candidates for sensing applications. Researchers have perform quantities of studies on the sensing properties of MoS_2_ ultrathin films to many kinds of chemical gas, such as NH_3_, NO, NO_2_, etc. [23–30]. These gas molecules belong to polar structures and charges can be exchanged easily between the surface of MoS_2_ and the above molecules. Thus, MoS_2_-based devices exhibit high sensing performance to the pole molecules, such as high sensitivity, ultra-low detective limit, and high-speed response. However, it is very difficult for H_2_ to be detected by MoS_2_ due to its nonpolar nature. Decorating MoS_2_ nanosheets with metal palladium (Pd) nanoparticles can increase the sensor response and, especially, the Pd-decorated MoS_2_ composites have shown obvious response to H_2_ due to Pd catalytic effect [31, 32]. However, the H_2_ sensitivity of the reported Pd-decorated MoS_2_ sensors is low. In our previous studies [33, 34], we proposed heterojunction-type H_2_ sensor devices by combining MoS_2_ films with Si. As well known, Si is dominating the commercial electronic device market due to its high abundance and mature processing technology. It supplies a simple route to develop practically applicable devices through the integration of MoS_2_ onto Si [35–38]. Our results demonstrate that the MoS_2_/SiO_2_/Si heterojunctions as H_2_ sensors exhibit high sensitivity, about 10^4^%. However, the response and recovery time is very long, ~ 443.5 s. The slow response speed is mainly caused by the difficulties of the H diffusion in the thick films. Based on the above analysis, high-sensitive performance would be realized through the integration of 2D few-layer MoS_2_ films onto Si wafers. To our knowledge, no related results are presented previously.

In this work, we report the growth of wafer-scale, few-layer MoS_2_ ultrathin films onto SiO_2_/Si using DC sputtering technique and the surface decoration of the MoS_2_ are performed by the synthesis of Pd nanoparticles. Moreover, the Pd-decorated MoS_2_/SiO_2_/Si heterojunctions show obvious electrical response toward H_2_ and the performance can be featured by a high sensitivity, fast response, and recovery. The effect of the thickness of the Pd layer on H_2_ sensing performance is further studied. The sensing mechanism is clarified by the construction of the energy-band alignment at the interface of the fabricated heterojunction.

## Methods

Few-layer MoS_2_ films were grown on (100)-oriented Si substrates by DC magnetron sputtering technique. The homemade polycrystal MoS_2_ target was used in this work, and its purity was about 99%. The Si substrates used in this work were n-type semiconductors, and the resistivity is about 1–6 Ω cm. Before the deposition, the wafers were cut into 12.5 mm × 12.5 mm slices and ultrasonically cleaned in sequence by alcohol, acetone, and de-ionized water. Then, the substrates were dipped into HF solution (~ 5%) for 60.0 s to remove the natural oxide layer from the Si surface. After that, oxidation treatment of the substrates were performed in peroxide solution (~ 40.0%) at 100 °C for 20.0 min to form a SiO_2_ passivation layer on the Si surface. The SiO_2_ layer had two roles in the heterojunction. The SiO_2_ layer could supply smooth substrate surface for the 2D layer-mode growth of the MoS_2_ layers. Simultaneously, the SiO_2_ layer could improve the interface of the MoS_2_/Si by decreasing the diffusion between the MoS_2_ and Si. Subsequently, the MoS_2_ films were grown on the SiO_2_-buffered Si substrates at the temperature of 450 °C, respectively. During the deposition, the pressure of argon gas and working power was kept at 1.0 Pa and 10.0 W, respectively. After the growth of the MoS_2_ films, the Pd-nanoparticle layers with different thickness (1.0, 3.0, 5.0, 10.0, 15.0, and 30.0 nm) were sputtered in situ and decorated onto the MoS_2_ surface, respectively. The deposition temperature, working pressure, and power were RT (~ 300 K), 3.0 Pa, and 10.0 W, respectively. Finally, about 300-μm-thickness Indium (In) pads with a diameter of 0.5 mm as electrodes were pressed on the surface of the Pd-decorated MoS_2_ films and the Si backside as the electrodes, respectively.

MoS_2_ films were characterized using Raman spectroscopy (HORIBA, HR800) with the excitation wavelength of 488 nm. The surface of the sample was characterized by atomic force microscope (AFM). X-ray photoemission spectroscopy (XPS) spectra were performed by a Kratos Axis ULTRA spectrometer using a monochromatic Al Kα X-ray source (1486.6 eV). High-resolution transmission electron microscopy (HRTEM) was performed on a JEOL JEM-2100F. The transmission spectra were measured by Shimadzu UV-3150 spectrophotometer. Ultraviolet photoelectron spectroscopy (UPS) was performed using an unfiltered He-I (21.22 eV) gas discharge lamp.

By the exposure of the sensors upon different concentration of H_2_ in dry air at RT, sensing properties were measured in a chamber, where the sensor device is mounted and the current was recorded by Keithley 2400 source meter. For the recovery of the sensors, the chamber was opened and air was filled into the chamber.

## Results and Discussion

**Fig. 1:**
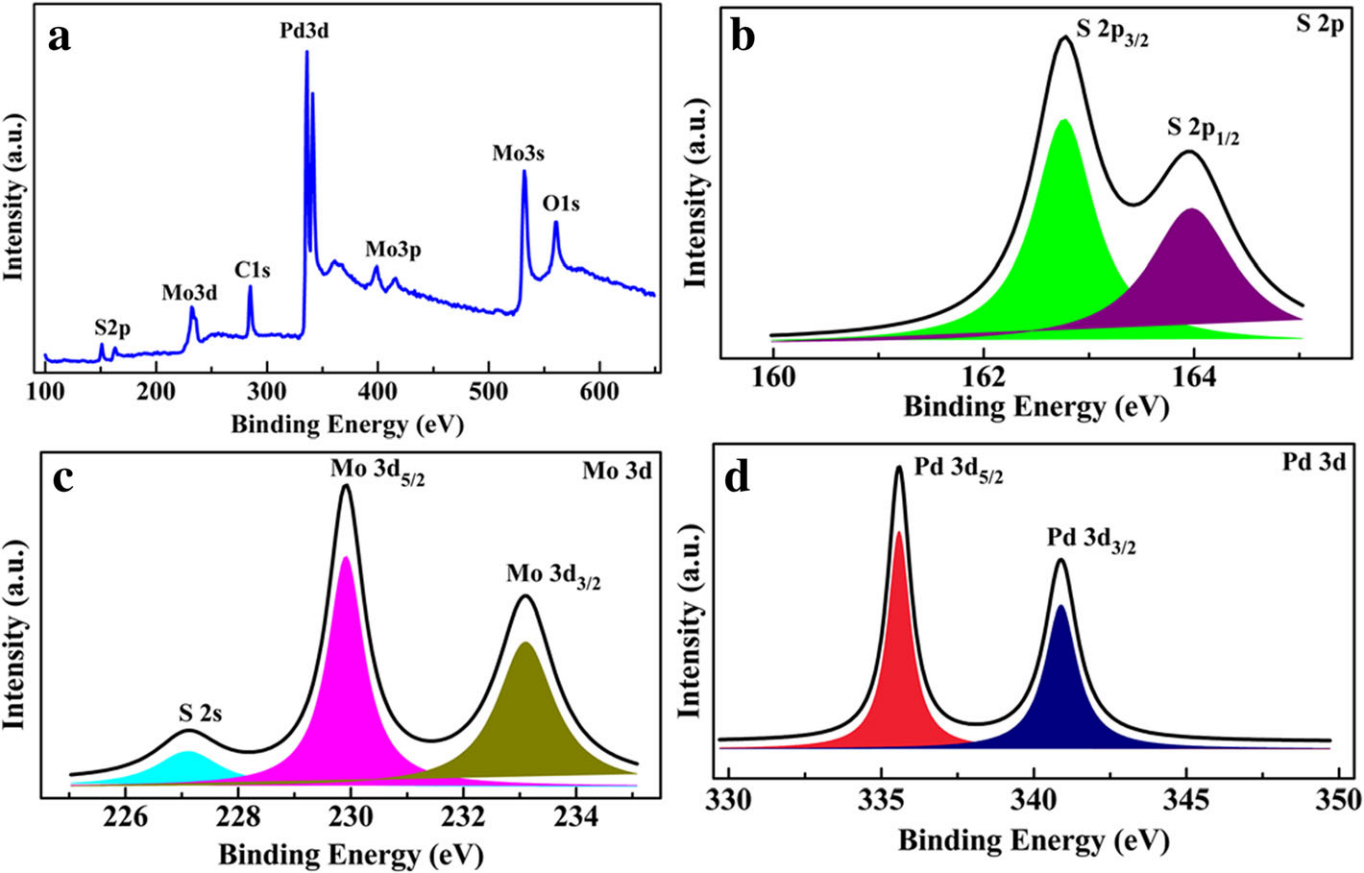
**a** XPS survey, **b** S 2p, **c** Mo 3d, and **d** Pd 3d core level spectra of the 5-nm Pd-decorated MoS_2_ layers on Si substrate

The chemical identification of Pd-decorated MoS_2_ film is conducted by XPS, as shown in Fig. 1. XPS survey spectrum of the sample is displayed in Fig. 1a. The spectrum consists of Mo, S, Pd, and O peaks, which implies successful synthesis of MoS_2_ with a small amount of residual carbon on the SiO_2_/Si substrate. In addition, the C peak might be caused by the residual gas during the deposition. As shown in Fig. 1b, the peaks at 163.9 and 162.8 eV correspond to the S 2p_1/2_ and 2p_3/2_, respectively. The Mo 3d core level spectrum is shown in Fig. 1c. The Mo 3d_3/2_ and 3d_5/2_ peaks of the MoS_2_ layer are located at the 233.1 and 229.9 eV, respectively. Additionally, the S 2s peak is appeared at 227.1 eV. These results are almost in accord with other results [39, 40], suggesting that the sputtered MoS_2_ layers have good chemical stoichiometry. As shown in Fig. 1d, two peaks at 340.9 and 335.5 eV are assigned to Pd 3d_3/2_ and 3d_5/2_, respectively. The binding energies are similar with those for Pd metals [41], demonstrating that the few-layer MoS_2_ is covered by the metal Pd layer and no obvious substitution of Mo atoms with the Pd doping happens.

**Fig. 2:**
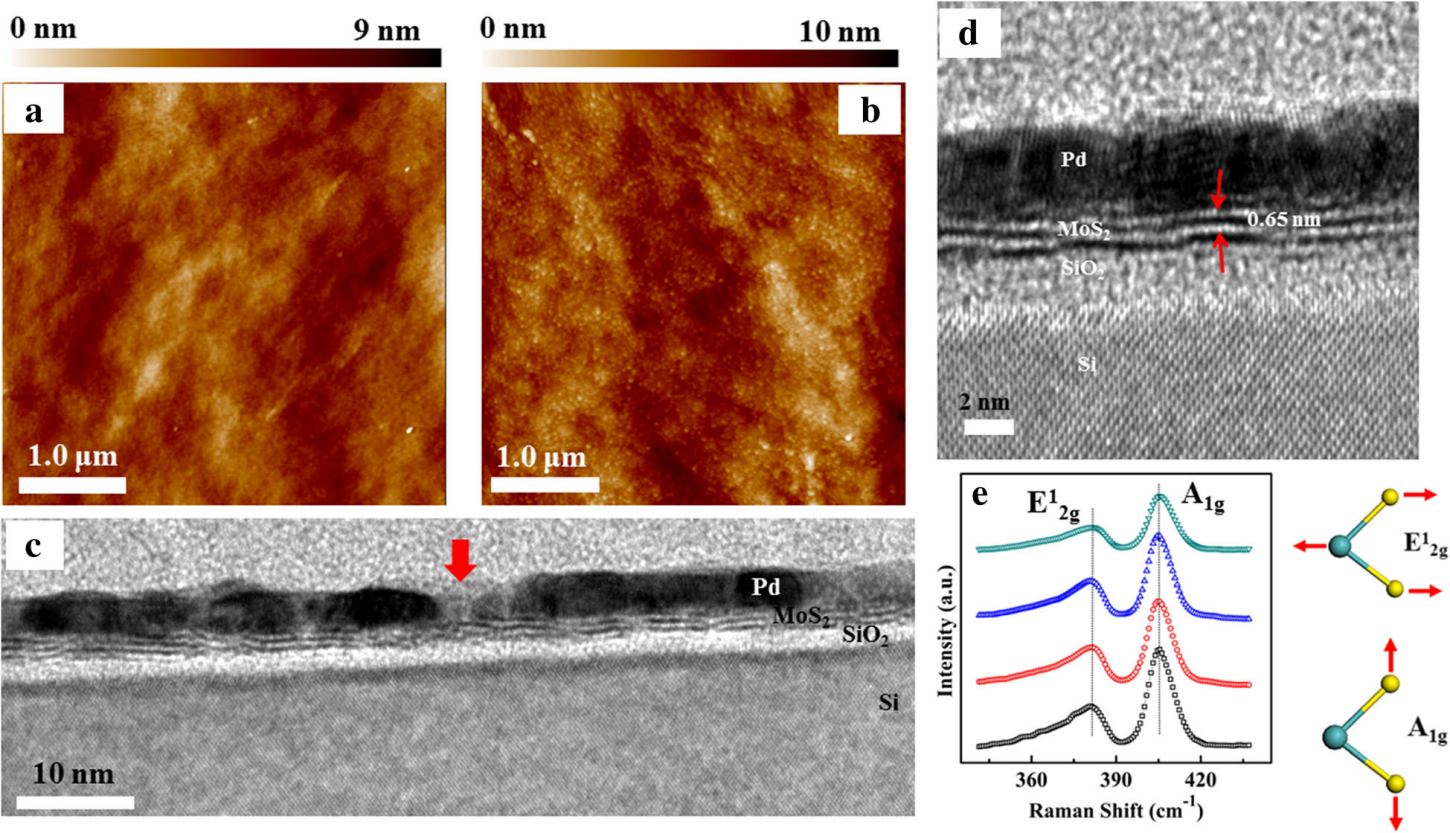
AFM images of the MoS_2_ layers **a** without Pd decoration, and **b** with 5-nm Pd decoration. **c** HRTEM image of the Pd-decorated MoS_2_ layers on the Si substrate. The red arrow denotes the gap between two Pd nanoparticles. **d** Enlarged HRTEM image. **e** Typical Raman spectra of the as-grown MoS_2_ layers on Si from different regions of the sample

Figure 2a shows the AFM images of the few-layer MoS_2_. The MoS_2_ layer has smooth surface and no obvious outgrowth are observed, demonstrating the 2D-mode growth of the MoS_2_ film. According to our results, the root-mean-square roughness (RMS) is about 0.78 nm. After the deposition of the 5-nm Pd decoration layer, quantities of nanoparticles on the surface can be seen clearly, as shown in Fig. 2b. This implies the island-like 3D-mode growth of the Pd layer. The average diameter of the Pd nanoparticles is about 47.7 nm, and the surface roughness slightly increased to 0.89 nm due to the deposition of the Pd nanoparticles. Additionally, the size of the Pd nanoparticles shows obvious dependence on the Pd deposition thickness (Additional file 1: Figure S1). Figure 2c shows the cross-sectional HRTEM image of the Pd-decorated MoS_2_ layers on the Si substrate. The Pd layer and MoS_2_ film can be seen clearly from the figure. From the figure, obvious gap of ~ 7.2 nm in the Pd layer can be seen, as denoted by the red arrow in the figure. This suggests that the 5-nm Pd layer is discontinuous and large quantities of Pd nanoparticles are formed on the MoS_2_ surface. The sputtered MoS_2_ film shows clear layered structure with 2–3 atomic S–Mo–S layers and the distance between the unit layers is about 0.65 nm, as shown in the enlarged HRTEM image in Fig. 2d. In order to further illustrate the homogeneity, Raman spectra of the few-layer MoS_2_ are taken from four different regions of the sample, respectively. Regardless of the location, two typical Raman active modes of MoS_2_ can be seen from Fig. 2e, the E^1^_2g_ mode at ∼ 381.9 cm^−1^ and A_1g_ mode at ∼ 405.1 cm^−1^. The E^1^_2g_ mode corresponds to the sulfur and molybdenum atoms oscillating in antiphase parallel to the crystal plane and the A_1g_ mode corresponds to the sulfur atoms oscillating in antiphase out-of-plane, as shown in the right insets. The difference of the Raman shifts between the A_1g_ and E^1^_2g_, ~ 23.2 cm^−1^ reflects the number of MoS_2_ layers. This value is larger than the monolayer MoS_2_ [42–44], while smaller than the bulk [45–47], indicating the synthesis of few-layer MoS_2_.

**Fig. 3:**
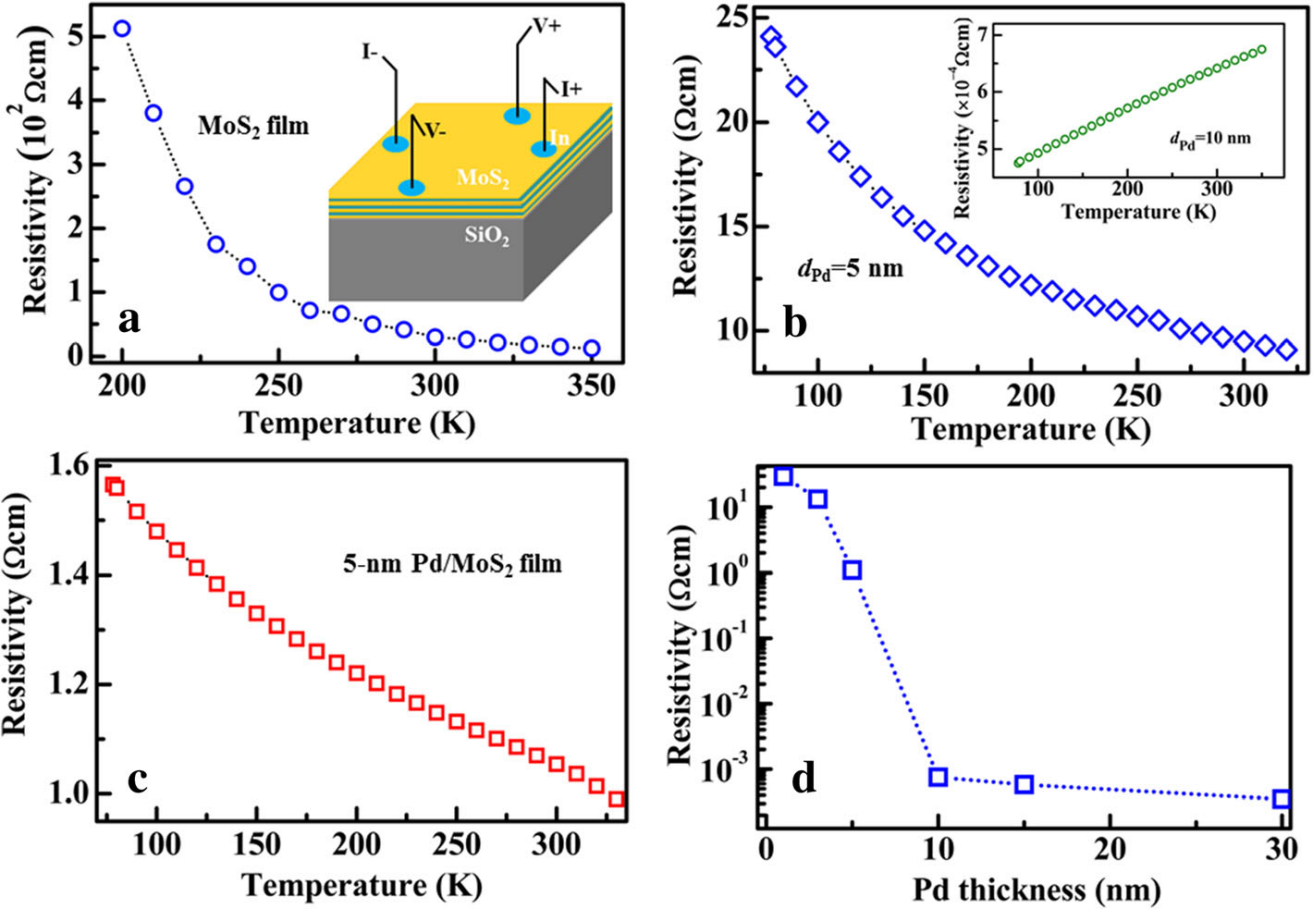
Resistivity-temperature curve of different samples grown on 300-nm SiO_2_/Si substrate. **a** Few-layer MoS_2_. The inset shows the schematic illustration for the measurements. **b** 5-nm Pd layer. The inset shows the *ρ*-*T* curve for the 10-nm Pd layer. **c** 5-nm Pd-decorated MoS_2_ layers. **d** Resistivity of the Pd-decorated MoS_2_ layers as a function of Pd thickness

In order to demonstrate the transporting characteristics of the Pd-decorated MoS_2_ films, the dependence of the resistivity (*ρ*) on temperature (*T*) of different samples grown on 300-nm SiO_2_/Si substrates were investigated, as shown in Fig. 3. Figure 3a shows the *ρ*-*T* curve for the few-layer MoS_2_ and the inset shows the schematic illustration for the measurements using van der Pauw technique. The resistivity of the MoS_2_ decreases with increasing the measurement temperature, which is in accord with its semiconductor nature. Figure 3b shows the *ρ*-*T* curve of the 5-nm Pd layer and the inset shows the *ρ*-*T* curve of the 10-nm Pd layer. Due to the discontinuity, the resistivity of the 5-nm Pd layer decreases with increasing the temperature, showing the semiconductor characteristics. When the Pd layer increases to 10 nm, the resistivity increases with increasing the temperature, as shown in the inset. This is in accord with the metal characteristics, implying that the Pd layer becomes continuous when the Pd increases from 5 to 10 nm. When the few-layer MoS_2_ is decorated by 5-nm Pd, its *ρ*-*T* curve is shown in Fig. 3c. The Pd-decorated MoS_2_ film exhibits semiconductor characteristics, and its resistivity decreases with increasing the temperatures. Moreover, the resistivity for the Pd-decorated MoS_2_ film is about 1.1 Ω cm. This value is much smaller than those for single MoS_2_ layer and 5-nm Pd layer, 29.6 and 9.5 Ω cm, respectively. The large decrease of the resistivity of the Pd-decorated MoS_2_ film must be induced by the effective connection between the Pd layer and the few-layer MoS_2_ at the interface. Figure 3d further shows the dependence of the resistivity of the Pd-decorated MoS_2_ film on the thickness of the Pd layer (*d*_*Pd*_). The resistivity of the Pd-decorated MoS_2_ films decreases with increasing the Pd thickness, and the sharp decrease of the resistivity is observed when *d*_*Pd*_ > 5 nm. This means that the discontinuous Pd nanoparticles reach the maximum coverage on the surface of the few-layer MoS_2_ when *d*_*Pd*_ is around 5 nm.

**Fig. 4:**
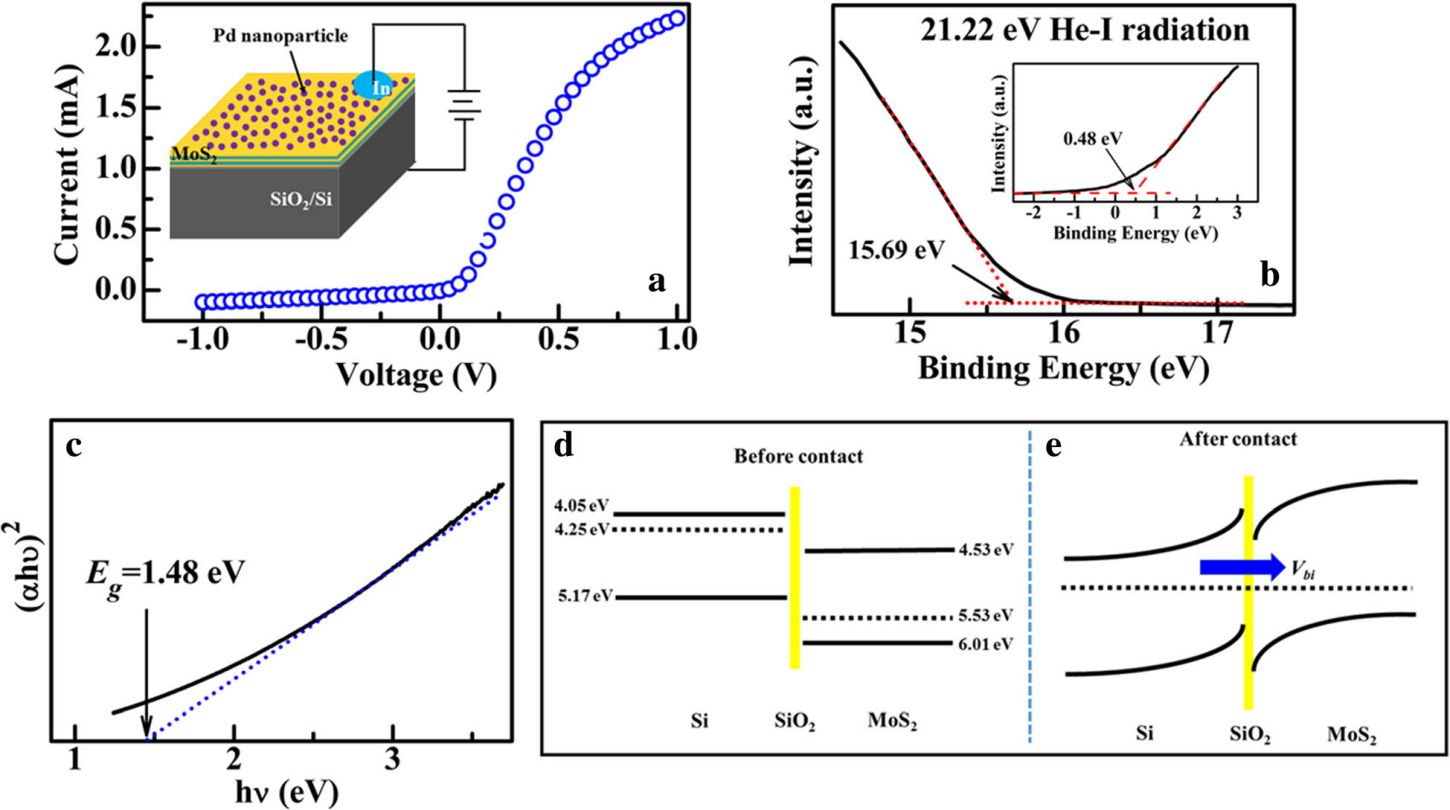
**a** I-V characteristics of Pd-decorated MoS_2_/Si heterojunction. The inset shows the schematic illustration for the measurements. **b** UPS spectra of the MoS_2_ layers on Si substrate. **c** Curve of (*αhν*)^2^ vs *hν* of the MoS_2_ layers. The energy band diagram at the MoS_2_/Si interface before contact (**d**) and after contact (**e**)

Figure 4a shows the I-V curve of the Pd-decorated MoS_2_/SiO_2_/Si junction at room temperature and *d*_*Pd*_ = 5.0 nm. The inset shows the schematic illustration for the measurements. From the figure, the junction exhibits obvious rectifying behavior. Figure 4b shows the UPS spectrum of the few-layer MoS_2_ film. The work function (*W*) of the film is calculated by the difference between the cutoff of the highest binding energy and the photon energy of the exciting radiation [48], ~ 5.53 eV. The distance (*E*_*p*_) between the valence band (*E*_*V*_) and Fermi level (*E*_*F*_) of the MoS_2_ film is extracted from the onset energy, as shown in the inset, ~ 0.48 eV. From the transmission spectrum of the MoS_2_ film (Additional file 1: Figure S2), (*αhν*)^2^ is plotted as a function of photon energy *hν* in Fig. 4c, wherein *h*, *ν*, and *α* represent the Planck constant, photon frequency, and the absorption coefficient, respectively [49]. The band gap (*E*_*g*_) of the film is determined by the intercept of the line on *hν* axis, *E*_*g*_ = 1.48 eV. Accordingly, the *p*-type behavior for the as-grown MoS_2_ film can be proved. Hall measurements further shows that the concentration of the hole-type carrier and the mobility are about 4.38 × 10^15^/cm^3^ and 11.3 cm^2^/Vs, respectively. The *p*-type characteristics might be caused by the adsorption of other gas molecules [39]. Based on above results, the isolated energy-band diagrams of the few-layer MoS_2_ film and *n*-Si are constructed, as shown in Fig. 4d. In the figure, *W* = 4.21 eV, *E*_*g*_ = 1.12 eV, and *E*_*p*_ = 0.92 eV for *n*-Si are used [50]. Additionally, the SiO_2_ layer as the surface passivation layer of the Si substrate is incorporated into the interface in the energy-band diagram. When the Pd-decorated MoS_2_ film is deposited onto the Si substrate, the electrons flow from the substrate into the film at the interface due to the higher *E*_*F*_ of the Si. The flowing process stops when the Fermi levels are equal and the Pd-decorated MoS_2_/Si *p-n* junction is fabricated, as shown in Fig. 4d. As a result, a built-in electrical field (*V*_*bi*_) is formed near the interface and its direction points from the substrate to the MoS_2_. Thus, asymmetric characteristics and obvious rectifying characteristics can be observed from the I-V curve in Fig. 4a. In a semiconductor heterojunction [51], the reverse current can be described as

1 $$ {I}_{-}\propto \exp \left(-\frac{qV_{bi}}{k_0T}\right) $$

where *I*_*−*_, *q*, *k*_*0*_, and *T* represent the reverse current, electron charge, Boltzmann constant, and temperature. Thus, the currents of the Pd-decorated MoS_2_/Si *p-n* junction could be changed by tuning the built-in field *V*_*bi*_.

**Fig. 5:**
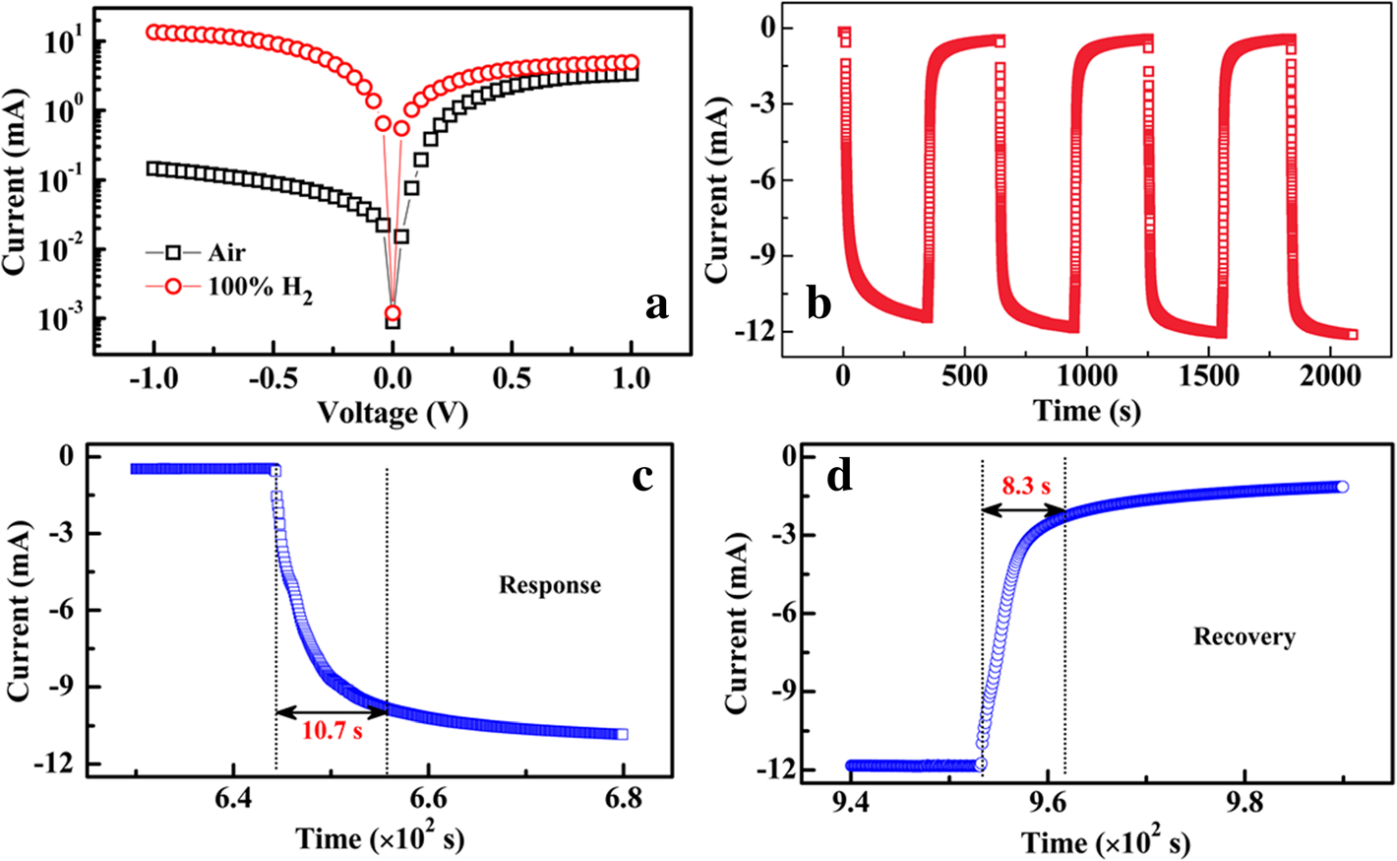
**a** LgI~V curves of the Pd-decorated MoS_2_/Si heterojunction. **b** *I* vs *t* graphs of the Pd-decorated MoS_2_/Si heterojunction exposed to pure H_2_ at − 1.0 V and RT. **c**, **d** Enlarged response and recovery edges, respectively, of the sensing curve. The response time (*t*_res_) is the time interval for the response to rise from 10 to 90% of the total current change. The recovery time (*t*_rec_) is the time interval for the response to decay from 90 to 10% of the total current change

Figure 5a shows the semi-logarithm plot of measured I-V curves of the Pd(5.0 nm)-decorated few-layer MoS_2_/SiO_2_/Si *p-n* junction in air and pure H_2_ at RT, respectively. From the figure, obvious H_2_ sensing characteristics can be seen in the reverse voltage range. The sensitivity (*S*) of the device is defined as

2 $$ S=\frac{I_{H2}}{I_{\mathrm{air}}}\times 100\% $$

where *I*_*H2*_ and *I*_air_ represent the current under H_2_ and air condition, respectively. At − 1.0 V, *S* is calculated, ~ 9.2 × 10^3^%. This value is much larger than the result (only 35.3%) of the single Pd-decorated few-layer MoS_2_ sensor [32]. Comparatively, the sensitivity of few-layer MoS_2_/SiO_2_/Si heterojunction without Pd decoration and 5-nm Pd/SiO_2_/Si heterojunction without the few-layer MoS_2_ in our experiments are just 15 and 133% (Additional file 1: Figure S3), respectively. Thus, the H_2_ sensing characteristics can be enhanced greatly due to the effective connection between the Pd-nanoparticles and few-layer MoS_2_. When the Pd-decorated MoS_2_ is exposed upon H_2_, the Pd nanoparticles as the sensitive layer reacts with hydrogen molecules and palladium hydride (PdH_x_) is formed [52]. Consequently, large quantities of electrons are released from the Pd layer and injected into the MoS_2_ film, resulting that the hole-type carriers are compensated and the hole concentration decreases. This can cause the shift of the Fermi level of the MoS_2_ film toward the conduction band accordingly and the barrier height induced by the *V*_*bi*_ at the MoS_2_/Si interface decreases. According to Eq. 2, the junction currents increase after the device exposure to H_2_. When the heterojunction is biased positively, the sensing characteristics are much poorer than that in negative bias range, as shown in the figure. In the positive voltage range, large quantities of electrons are injected into the MoS_2_ layers from the Si substrate. Under this condition, the electrons from PdH_x_ have little effects on the electron concentration of the MoS_2_ layer. Thus, the heterojunction shows unobvious sensing characteristics in the positive range. Figure 5b presents the reproducible current change of the Pd-decorated MoS_2_/SiO_2_/Si sensor in H_2_ conditions at − 1.0 V and RT. When the conditions are changed alternately between air and H_2_, two distinct current states for the sensor are shown, the “high” current state in air and the “low” current state in H_2_, respectively. As shown in the figure, both the “high” and “low” states are stable and well reversible. The response and recovery speeds are evaluated by the rise and fall edges, respectively, of the sensing curve, as shown in Fig. 5c, d. The response time (*t*_res_) is defined as the time interval for the current to rise from 10 to 90% of the total change and the recovery time (*t*_rec_) is the time interval for the current to decay from 90 to 10% of the total change. From the figures, the response and recovery of 10.7 and 8.3 s, respectively, can be estimated. It is worth noting that the fast response and recovery of the Pd-decorated MoS_2_/SiO_2_/Si sensor is one of the best results achieved for the H_2_ sensors at RT [2–5]. During the sensing process, the few-layer MoS_2_ is crucial based on the following three aspects: (i) 2D MoS_2_ layer provides high surface-to-volume ratio and serves as a platform for the connection of the Pd nanoparticles, which could promise the sensor highly sensitive characteristics to H_2_ exposure. (ii) Layered structure supplies large storage space for the electrons injected from Pd nanoparticles. This can enhance significantly the sensitivity of the fabricated sensor. In contrast, in a monolayer graphene sensors, the sensitivity could be limited by the low storage space of the injected carriers. (iii) As shown in Fig. 2c, d, due to its continuous characteristics, the MoS_2_ layer offers high-speed paths for the transporting of the injected carriers. Thus, a high response and recovery speeds can be achieved.

**Fig. 6:**
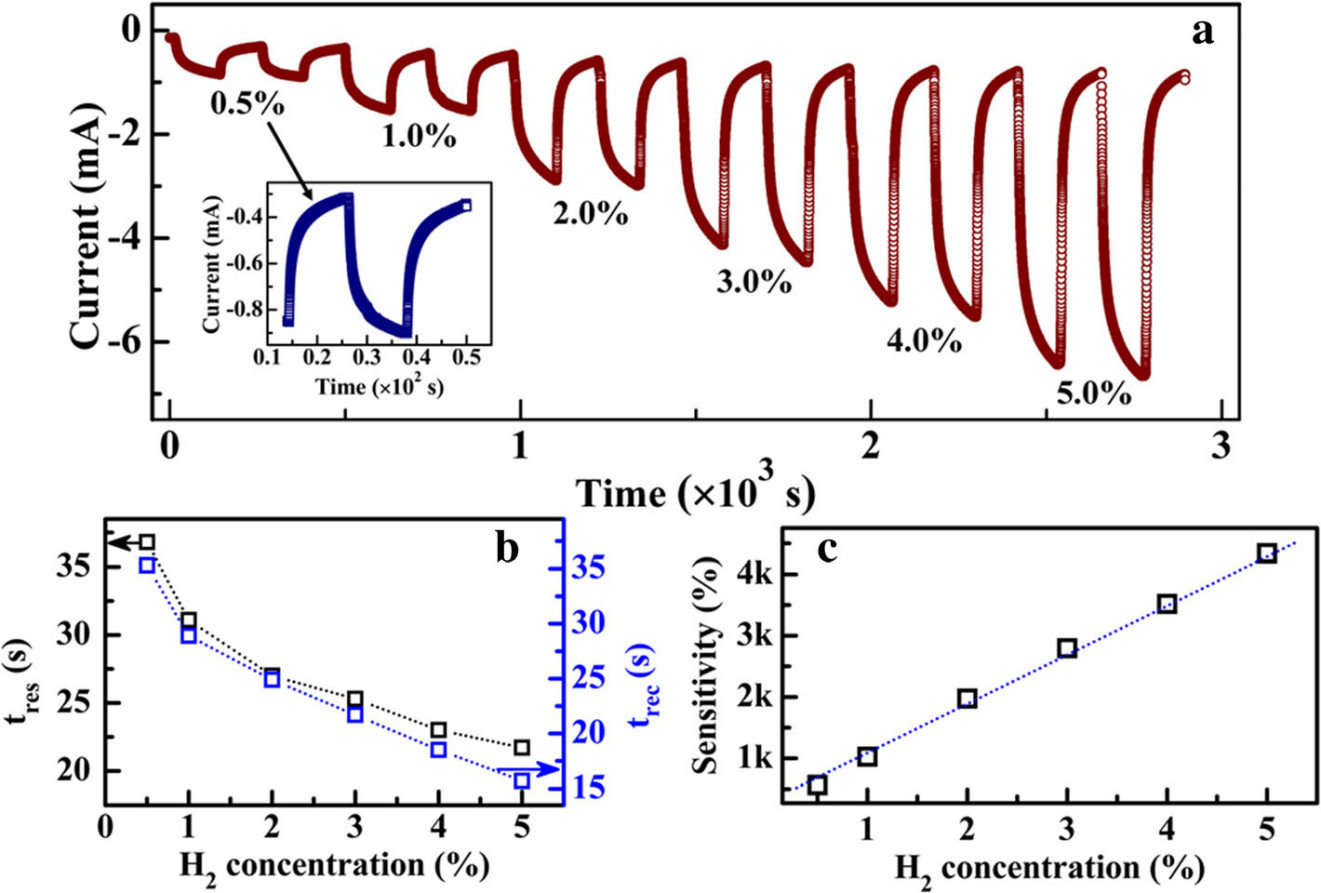
**a** Dynamic responses of the Pd-decorated MoS_2_/Si heterojunction upon consequent H_2_ at varying concentrations from 0.5 to 5% at − 1.0 V. The inset shows the enlarged image of the sensing characteristics of the heterojunction under H_2_ of 0.5%. **b** Dependence of *t*_res_ and *t*_rec_ on H_2_ concentration. **c** Dependence of the sensing response of the heterojunction on H_2_ concentration

Figure 6a shows the dynamic response of the Pd-decorated MoS_2_/SiO_2_/Si sensor upon varying H_2_ concentration from 0.5 to 5.0% at − 1.0 V. The inset shows the enlarged sensing curve of the sensor upon the H_2_ concentration of 0.5%. The sensor exhibits significant response at each H_2_ level, even at the low concentration of 0.5%. Strong dependence of the response on H_2_ levels can be seen from the figure. Figure 6b further shows the response and recovery time as a function of H_2_ concentration, respectively. As shown in the figure, both *t*_res_ and *t*_rec_ increase continuously with decreasing the H_2_ levels. When the H_2_ concentration decreases from 5.0 to 0.5%, *t*_res_ increases from 21.7 to 36.8 s and *t*_rec_ increases from 15.5 to 35.3 s. Figure 6c shows the dependence of the sensitivity of the sensor upon H_2_ levels. The sensor shows an almost linear correlation between its sensitivity and the H_2_ concentration. When the sensor is exposed to H_2_ concentration of 5%, *S* is about 4.3 × 10^3^%. With decreasing the H_2_ level, *S* decreases gradually, which is caused by the reduction of the reduced amount of hydrogen molecules absorbed by the Pd nanoparticles. Under the H_2_ concentration of 0.5%, *S* decreases to 5.7 × 10^2^%.

**Fig. 7:**
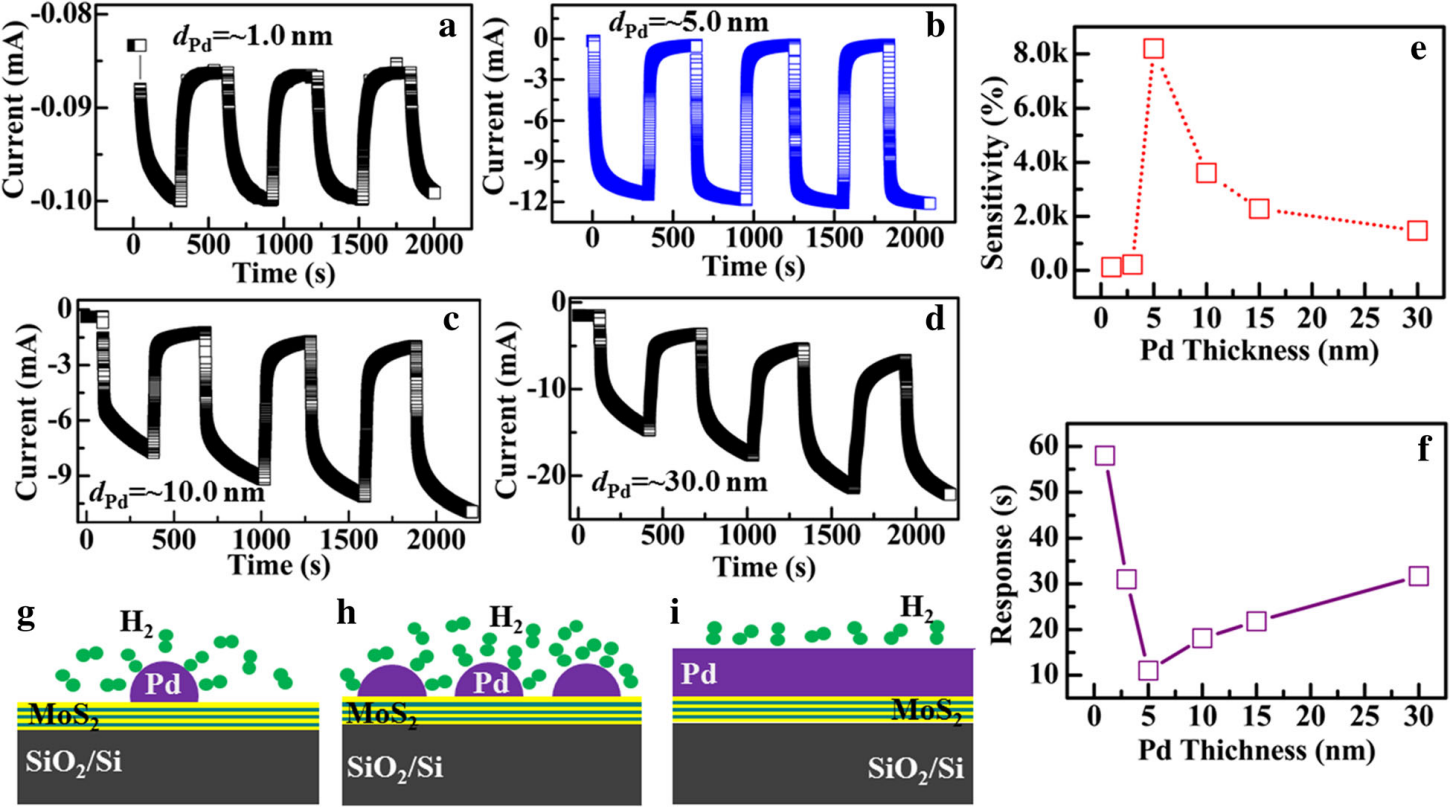
Sensing characteristics of the Pd-decorated MoS_2_/Si heterojunction with different Pd thickness, respectively. **a** *d*_*Pd*_ = ~ 1.0 nm, **b** *d*_*Pd*_ = ~ 5.0 nm, **c** *d*_*Pd*_ = ~ 10.0 nm, and **d** *d*_*Pd*_ = ~30.0 nm. **e**, **f** Dependence of the sensitivity and response time of the heterojunctions on Pd thickness, respectively. **g**–**i** Schematic illustration of the reaction of H_2_ on the Pd-decorated MoS_2_/Si heterojunction with different Pd thickness

The Pd thickness is a crucial factor to control the density of Pd nanoparticles and further determine the sensing performance. Figure 7a–d shows the sensing curves of Pd-decorated MoS_2_/SiO_2_/Si sensors with different Pd thickness, *d*_*Pd*_ = ~ 1.0, ~ 5.0, ~ 10.0, and ~ 30.0 nm. As shown in the figure, each sensor shows obvious sensing characteristics to H_2_. Figure 7e, f shows the sensitivity and response time of the sensors as a function of the Pd thickness. Figure 7g–i shows the schematic illustration of the reaction of H_2_ on the Pd-decorated MoS_2_/SiO_2_/Si heterojunction with different Pd thickness. When the Pd layer is very thin, such as 1.0 nm, the Pd particles on the few-layer MoS_2_ are unclear (Additional file 1: Figure S1) and the coverage density of the Pd nanoparticles might be very low, as shown in Fig. 7g. Under this condition, the sensor shows the sensing characteristics to H_2_, however, the resulted sensitivity is only 120.7% and the response is relatively slow, about 58.1 s. With the Pd thickness increases, the coverage density of the Pd nanoparticles increases on the few-layer MoS_2_ surface, as illustrated in Fig. 7h. Large quantities of H_2_ molecules can react quickly with the Pd nanoparticles due to the increased contacting area and large quantities of electrons are released into the few-layer MoS_2_. Consequently, the sensitivity of the sensor gradually increases, as shown in Fig. 7e. When *d*_*Pd*_ = 5.0 nm, the sensor exhibits the maximum *S* value of ~ 9.2 × 10^3^% with a fast response of 10.7 s. Thus, the improved sensing characteristics can be attributed to the increased coverage of Pd nanoparticles. When the Pd thickness further increases, however, the sensitivity of the sensor decreases. In a thick Pd layer, such as *d*_*Pd*_ = 30.0 nm, the Pd layer becomes continuous and the amount of the Pd nanoparticles reduces largely, as shown in Additional file 1: Figure S1. This results in the decrease of the contacting area between the device surface and ambient H_2_, leading to the decrease of the sensitivity. When *d*_*Pd*_ = 30.0 nm, *S* = 1.5 × 10^3^%. From Fig. 7c, d, obvious charge accumulation that both the *I*_air_ and *I*_*H2*_ exhibit negative slopes over the total duration of the exposure can be seen in the sensing curves for the sensors with the thick Pd layer [47]. This is not obvious for the sensors with thinner Pd layers (*d*_*Pd*_ = 1.0, 3.0, and 5.0 nm). Due to the charge accumulation, the response time of the sensors increases when *d*_*Pd*_ > 5.0 nm, as shown in Fig. 7f. Thus, ~ 5.0 nm is the optimized Pd thickness for the sensor with the highest coverage of Pd nanoparticles to achieve the best sensing characteristics.

## Conclusions

In summary, few-layer MoS_2_ films were grown on Si substrates via DC magnetron sputtering technique and Pd nanoparticles are further synthesized on the MoS_2_ surface to promote the detection of H_2_. Due to the decoration of the Pd nanoparticles on the device surface, especially the unique microstructural characteristics and excellent transporting properties of the few-layer MoS_2_ film, the fabricated sensor exhibits a high sensitivity of 9.2 × 10^3^% in pure H_2_ with a fast response of 10.7 s and recovery of 8.3 s. Additionally, the H_2_ sensing properties of the sensors are dependent largely on the size of the Pd layer and ~ 5.0 nm is the optimized thickness for the Pd-decorated MoS_2_/SiO_2_/Si junction to obtain the best sensing properties. The results indicate that sputtered Pd-decorated few-layer MoS_2_ combined with SiO_2_/Si semiconductors hold great promise for the scalable fabrication of high-performance H_2_ sensors.

## Additional File

Additional file 1: Figure S1. AFM images of the Pd-decorated MoS_2_ films with the Pd thickness of (a) *d*_*Pd*_ = 1 nm, (b) *d*_*Pd*_ = 3 nm, (c) *d*_*Pd*_ = 5 nm, (d) *d*_*Pd*_ = 10 nm, (e) *d*_*Pd*_ = 15 nm and (f) *d*_*Pd*_ = 30 nm. **Figure S2.** UV spectrum of the few-layer MoS_2_ film. **Figure S3.** Sensing curves of (a) the few-layer MoS_2_/SiO_2_/Si heterojunction and (b) 5-nm Pd/SiO_2_/Si heterojunction. (DOCX 1723 kb)

## Abbreviations

AFM: Atomic force microscope
*d*_*Pd*_: Thickness of the Pd layer
*E*_*C*_: Conduction band level
*E*_*F*_: Fermi energy level
*E*_*g*_: Energy band gap
*E*_*p*_: Distance between *E*_*V*_ and *E*_*F*_
*E*_*V*_: Valence band level
HRTEM: High-resolution transmission electron microscopy
MoS_2_: Molybdenum disulfide
RMS: Root-mean-square roughness
*t*_rec_: Recovery time for the sensor
*t*_res_: Response time for the sensor
UPS: Ultraviolet photoelectron spectroscopy
*V*_*bi*_: Built-in electrical field
*W*: Work function
XPS: X-ray photoemission spectroscopy
