# Supplementary material for: Highly Enhanced H2 Sensing Performance of Few-Layer MoS2/SiO2/Si Heterojunctions by Surface Decoration of Pd Nanoparticles
Source: Nanoscale Res Lett. 2017 Oct 17;12:567. doi: 10.1186/s11671-017-2335-y (PMC5645297; doi:10.1186/s11671-017-2335-y)
Supplement: Additional file 1: Figure S1. — AFM images of the Pd-decorated MoS2 films with the Pd thickness of (a) d Pd = 1 nm, (b) d Pd = 3 nm, (c) d Pd = 5 nm, (d) d Pd = 10 nm, (e) d Pd = 15 nm and (f) d Pd = 30 nm. Figure S2. UV spectrum of the few-layer MoS2 film. Figure S3. Sensing curves of (a) the few-layer MoS2/SiO2/Si heterojunction and (b) 5-nm Pd/SiO2/Si heterojunction. (DOCX 1723 kb) [file 11671_2017_2335_MOESM1_ESM.docx]

**Additional file 1**

**Highly enhanced H_2_ sensing performance of few-layer MoS_2_/SiO_2_/Si heterojunctions by surface decoration of Pd nanoparticles**


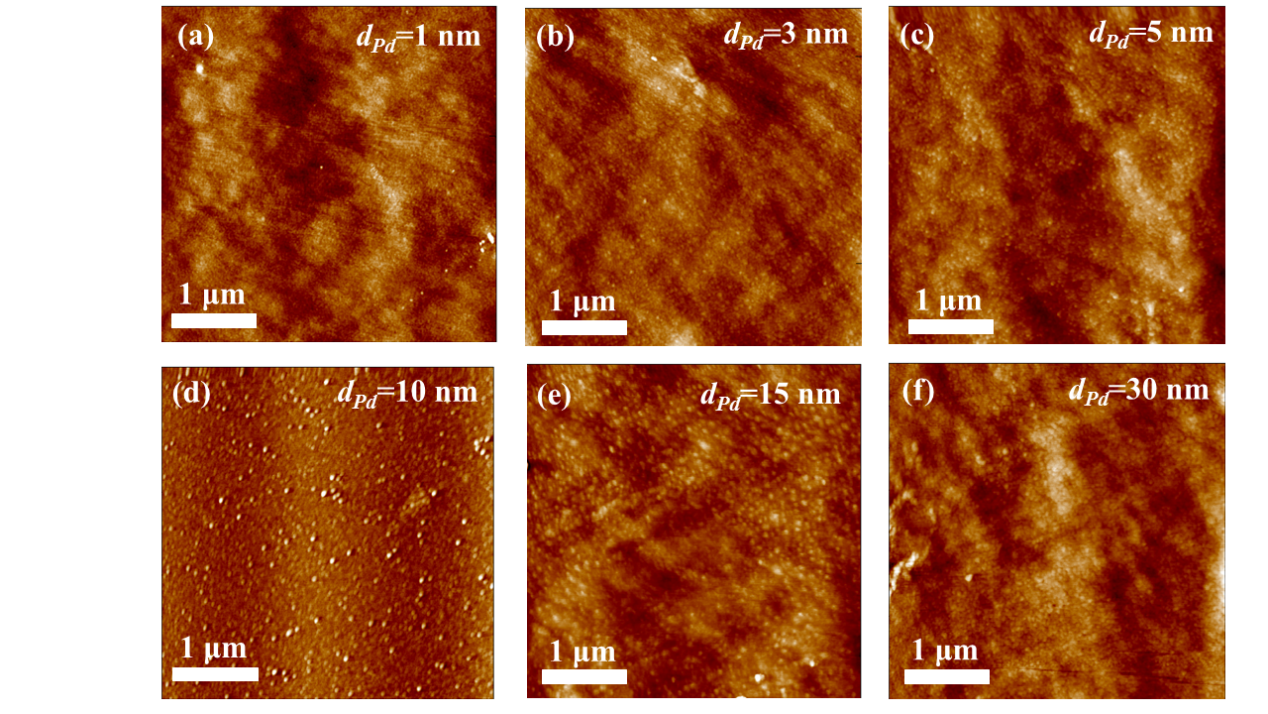


Figure S1. AFM images of the Pd-decorated MoS_2_ films with the Pd thickness of (a) *d_Pd_*=1 nm, (b) *d_Pd_*=3 nm, (c) *d_Pd_*=5 nm, (d) *d_Pd_*=10 nm, (e) *d_Pd_*=15 nm and (f) *d_Pd_*=30 nm.


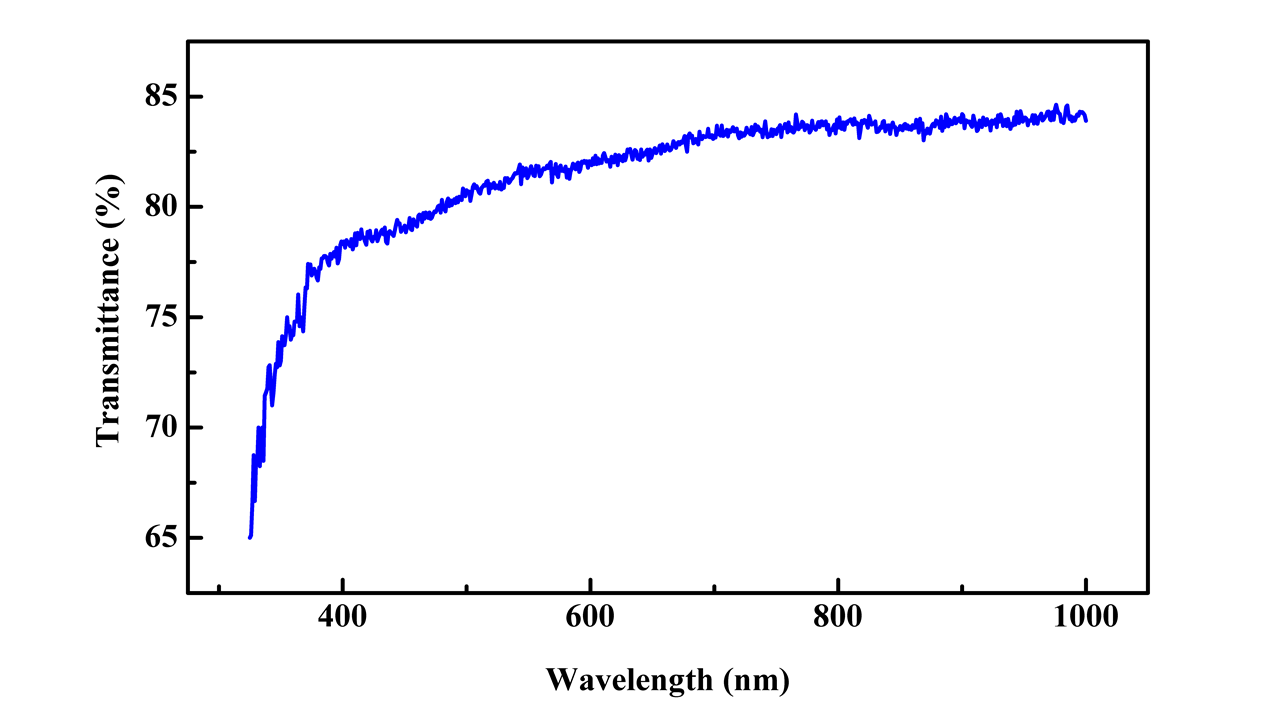


Figure S2. UV spectrum of the few-layer MoS_2_ film.


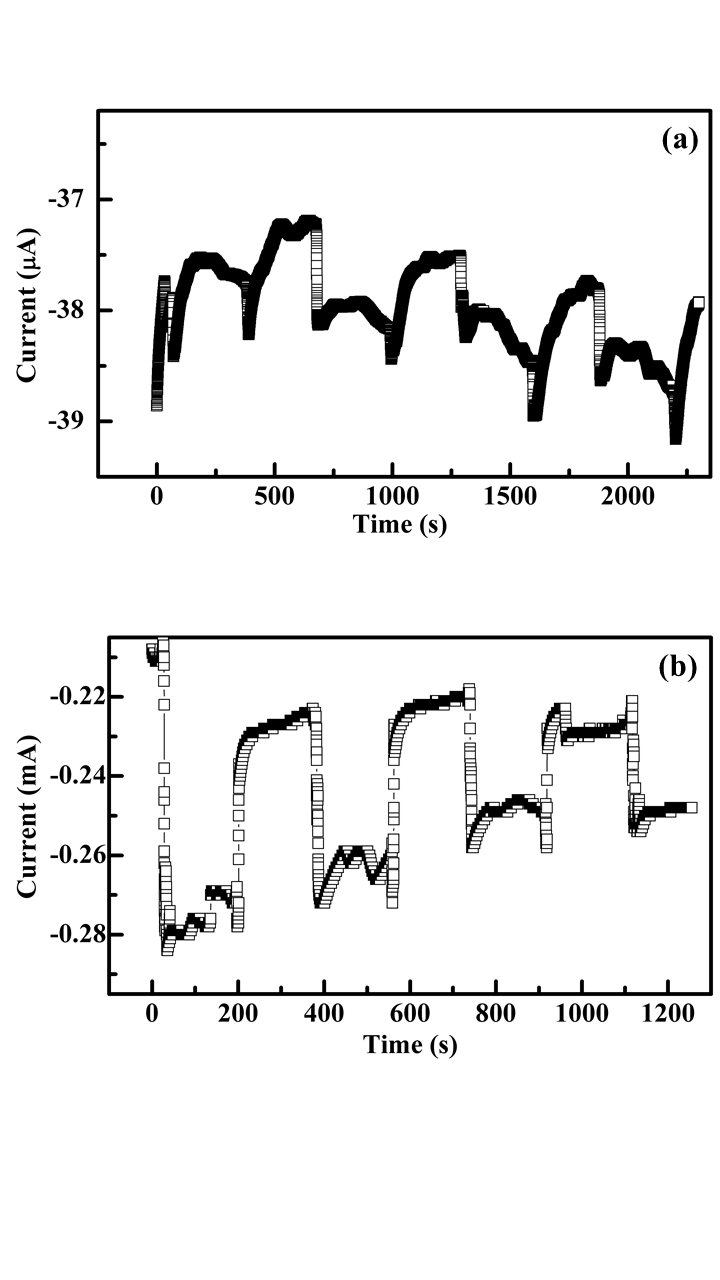


Figure S3. Sensing curves of (a) the few-layer MoS_2_/SiO_2_/Si heterojunction and (b) 5-nm Pd/SiO_2_/Si heterojunction.
